# Rare Variants in *PLXNA4* and Parkinson’s Disease

**DOI:** 10.1371/journal.pone.0079145

**Published:** 2013-11-11

**Authors:** Eva C. Schulte, Immanuel Stahl, Darina Czamara, Daniel C. Ellwanger, Sebastian Eck, Elisabeth Graf, Brit Mollenhauer, Alexander Zimprich, Peter Lichtner, Dietrich Haubenberger, Walter Pirker, Thomas Brücke, Benjamin Bereznai, Maria J. Molnar, Annette Peters, Christian Gieger, Bertram Müller-Myhsok, Claudia Trenkwalder, Juliane Winkelmann

**Affiliations:** 1 Neurologische Klinik und Poliklinik, Klinikum rechts der Isar, Technische Universität, München, Munich, Germany; 2 Institut für Humangenetik, Helmholtz Zentrum München, Munich, Germany; 3 Max-Planck Institut für Psychiatrie, Munich, Germany; 4 Munich Cluster for Systems Neurology (SyNergy), Munich, Germany; 5 Chair for Genome-Oriented Bioinformatics, Technische Universität München, Life and Food Science Center Weihenstephan, Freising-Weihenstephan, Germany; 6 Paracelsus Elena Klinik, Kassel, Germany; 7 Neurochirurgische Klinik, Georg August Universität, Göttingen, Germany; 8 Department of Neurology, Medical University of Vienna, Vienna, Austria; 9 Institut für Humangenetik, Technische Universität München, Munich, Germany; 10 Department of Neurology, Wilhelminenspital, Vienna, Austria; 11 Center for Molecular Neurology, Department of Neurology, Semmelweis University, Budapest, Hungary; 12 Institute for Epidemiology II, Helmholtz Zentrum München, Munich, Germany; 13 Institute for Genetic Epidemiology, Helmholtz Zentrum München, Munich, Germany; 14 Department of Neurology and Neurosciences, Stanford University, Palo Alto, California, United States of America; Centre Hospitalier Universitaire Vaudois (CHUV), Switzerland

## Abstract

Approximately 20% of individuals with Parkinson’s disease (PD) report a positive family history. Yet, a large portion of causal and disease-modifying variants is still unknown. We used exome sequencing in two affected individuals from a family with late-onset familial PD followed by frequency assessment in 975 PD cases and 1014 ethnically-matched controls and linkage analysis to identify potentially causal variants. Based on the predicted penetrance and the frequencies, a variant in *PLXNA4* proved to be the best candidate and *PLXNA4* was screened for additional variants in 862 PD cases and 940 controls, revealing an excess of rare non-synonymous coding variants in *PLXNA4* in individuals with PD. Although we cannot conclude that the variant in *PLXNA4* is indeed the causative variant, these findings are interesting in the light of a surfacing role of axonal guidance mechanisms in neurodegenerative disorders but, at the same time, highlight the difficulties encountered in the study of rare variants identified by next-generation sequencing in diseases with autosomal dominant or complex patterns of inheritance.

## Introduction

Characterized by resting-tremor, bradykinesia, rigidity, and postural instability, Parkinson’s disease (PD) is one of the most prominent neurodegenerative disorders. Genetic factors contribute significantly to the risk of developing PD–both sporadic and familial. Although up to 20% of PD cases are believed to be familial [1], [2], thus far, variants in only a few genes have been unequivocally shown to underlie familial PD. These include *PARK2*, *PINK1*, *PARK7*, *SNCA*, and *LRRK2* [3]–[8]. While all of these genes were identified by classical linkage analysis in large, multi-generation families, recently, next-generation sequencing has enabled the identification of disease-causing variants in smaller families and with an onset later in life without the need of genotypic information from more than one generation of affected individuals. By exome sequencing, *VPS35* was identified as a gene involved in late-onset familial PD [9], [10]. Still, to date, the identified genes only explain a small portion of the genetic “burden” in PD. However, a thorough understanding of the genetic alterations implicated in disease development is necessary to better comprehend disease pathogenesis and to provide more specific and, thus, more effective treatment options in the future.

Here, we describe exome sequencing of a German family with autosomal dominant late-onset PD in an attempt to pinpoint the disease-causing genetic variant.

## Methods

### Ethics Statement

Ethics review board approval was obtained from the ethics review board at Klinikum rechts der Isar, Technische Universität München, and Bayerische Landesärztekammer, both Munich, Germany, Hessische Landesärztekammer, Frankfurt, Germany, the ethics review board at Medical University Vienna, Vienna, Austria, and the ethics review board at Semmelweis University, Budapest, Hungary. Participants’ written informed consent was obtained.

### Participants

All living family members received a detailed neurologic exam by neurologists specializing in movement disorders. Cases and controls used in genotyping and variant screening have been reported previously [10], [11] and are described in more detail in the supplement.

### Exome Sequencing

Exome sequencing was performed with DNA isolated from lymphozytes of IV:11 and IV:18 on a Genome Analyzer IIx system (Illumina) after in-solution enrichment of exonic sequences (SureSelect Human All Exon 38 Mb kit for IV:11 and 50 Mb kit for IV:18, Agilent) as 76 bp paired-end runs. Read alignment was carried out with BWA (version 0.5.8). Single-nucleotide variants and small insertions and deletions (indels) were detected with SAMtools (version 0.1.7). Raw sequencing data are available upon request.

### Genotyping

All ten candidate variants tested for segregation by Sanger sequencing were genotyped in 975 cases and 1014 population-based controls pertaining to the KORA-AGE cohort using MALDI-TOF masspectrometry on the Sequenom® platform. Demographic data are given in the supplement. Association was tested by allelic statistics as implemented in PLINK.

### Linkage Analysis

We genotyped six family members (IV:11, IV:14, IV:16, IV:18, IV:20 and IV:21) with oligonucleotide SNP arrays (500 K, Illumina). Parametric linkage analysis was performed using a subset of 12,875 SNPs using MERLIN and an autosomal dominant model with incomplete penetrance of 70%.

### Variant Screening

We used Idaho®’s LightScanner high-resolution melting curve analysis to screen the coding regions and exon/intron boundaries of *PLXNA4* for variants. 862 cases and 940 population-based controls pertaining to the KORA-AGE cohort were included in the screening. Demographic data are given in the supplement. In the case of an altered melting pattern, Sanger sequencing ensued to identify the underlying variant. Group comparisons between cases and controls were performed for each gene and each variant separately using Fisher’s Exact and χ^2^ tests as appropriate.

### Cell Viability and Immunocytochemistry

Cultured primary fibroblasts from IV:11 and an offspring were stained using a live/dead staining (Invitrogen) and analyzed by FACS and stained with anti-PLXNA4 (1∶100, Sigma) and analyzed by fluorescence microscopy. Details are given in the supplement.

### Construction of a Qualitative Systems Biological Model

To investigate the role of *PLXNA4* in the PD biological system, we applied an integrative modeling approach to construct a qualitative multifactorial interaction network linking *PLXNA4* and genetic factors associated with PD. An interactome with known and predicted interactions of *PLXNA4* and its direct neighbors was prepared based on four commonly used databases and integrated to known PD pathways from KEGG and CIDeR as well as a manual literature search. For a detailed description see supplement.

## Results

### Pedigree and Clinical Phenotype

**Figure 1:**
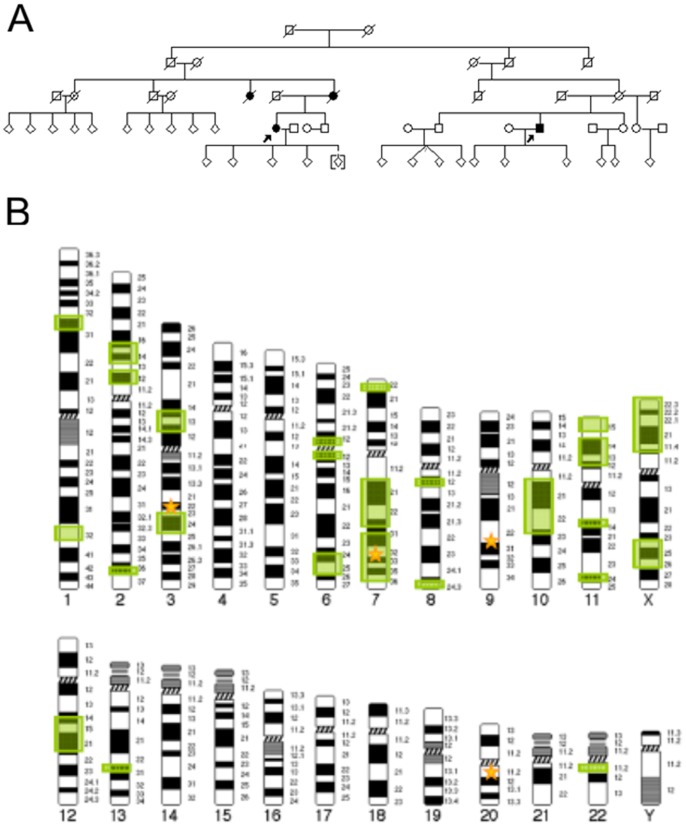
Pedigree and Linkage Analysis. (A) Pedigree of family used for exome sequencing. Open symbols indicate unaffected family members, affected individuals are denoted by closed symbols. An arrow denotes the individuals whose exomes were sequenced. Sex was obscured and birth order was altered to protect privacy. A diagonal line indicates a deceased individual. (B) 25 genomic regions on 12 chromosomes with logarithm of the odds (LOD) score≥0.5 were identified by linkage analysis. Green boxes represent genomic regions with LOD≥0.5, yellow stars represent the location of the four candidate genes remaining after frequency assessment (*GOLGA4*-chr3, *PLXNA4*-chr7, *OGN*-chr9, *CPNE1*-chr20). *PLXNA4* on chromosome 7 represents the only of the four genes overlapping a genomic region with LOD≥0.5.

We describe a five-generation family from Central Germany in which four members were affected by PD and the pattern of inheritance seems to be autosomal dominant with reduced penetrance (Figure 1). Clinical assessment revealed tremor-dominant, levodopa-responsive parkinsonism with an age of onset at 60 and 67 years of age in the two affected individuals examined (Table S1 in File S1). Both individuals also reported subjective cognitive impairment. Restless legs syndrome was present in IV:11 as well as one of her children. Transcranial ultrasound showed bilateral hyperechogenicity of the substantia nigra in IV:18 but was not performed in IV:11. MRI was in line with a diagnosis of PD in both. The affected parent (III:7) and aunt (III:5) of IV:11 were deceased before initiation of the study, so that no detailed phenotype information is available. Moreover, another aunt (III:2) on the same side of the family was reported to have suffered from an unclassified form of dementia.

### Identification of Candidate Variants by Exome Sequencing and Frequency Assessment of Candidate Variants in a Case/Control Cohort

Exome sequencing was performed using DNA from two second cousins (IV:11 and IV:18, Figure 1A). This generated 11.68 gigabases (Gb) of alignable sequence for IV:11 (average coverage = 108.46, base pairs with >8 reads = 93.67%) and 15.02 Gb for patient IV:18 (average coverage = 154.13, base pairs with >8 reads = 94.74%). All 28,803 detected variants shared by the two affected individuals were filtered against in-house exomes (n = 1739) of individuals with unrelated diseases. Here, variants were allowed to be present in ≤1% of exomes. Moreover, synonymous and non-coding variants as well as all variants annotated in dbSNP135 with a minor allele frequency (MAF) ≥0.01 were excluded from the follow-up (Figure S1). No known variants believed to play a causative role in PD were found in either IV:11 or IV:18.

All ten remaining missense, nonsense, stoploss, splice site or frameshift variants and indels were genotyped in 975 cases and 1014 population-based controls (Table 1). The variants were, overall, very rare. Two (*PLXNA4* p.Ser657Asn and *OGN* p.Leu124fs) were validated in the individual in whom they were first identified but were otherwise not found again in the 1989 individuals tested. *CPNE1* p.Ser1831Thr was present in the index case as well as one additional control individual and *GOLGA4* p.Gln425Arg was identified in one additional PD patient. The other six variants (*RBM28* p.Asp300Gly, *IMPDH1* p.His296Arg, *ARPP21* p.Ala576Thr, *PHF2* p.Ser840Asn, *SLC22A13* p.Arg16His and *SPANXE* p.Leu42Ile) were not as rare (MAF≥0.03%) and found at similar frequencies in both cases and controls and were, therefore, regarded to be unlikely candidates (Table 1).

**Table 1:** Ten Rare, Non-synonymous Variants Shared by Individuals IV:11 and IV:18 of Family PARK_0005.

Genomicposition (hg19)	gene	number of alleles	*in house* exomes (n = 1739)	genotyping		dbSNP135	1000genomes	NHLBI-ESP [39] (EA only)	transcript	variation		penetrance for PD in % (n = 6)	PolyPhen2
				cases (n = 975)	controls (n = 1014)					nucleotide	amino acid		
chr3∶35780947	*ARPP21*	1	12	9	11	rs151173813	0.0031∶2218	A = 37/G = 8563	NM_001267617.1	c.1726G>A	p.Ala576Thr	N/A	benign
chr3∶37365968	*GOLGA4*	1	4	2	0	rs139536585	not found	G = 8/A = 8592	NM_001172713.1	c.1274A>G	p.Gln425Arg	66.67%	benign
chr7∶127950857	*RBM28*	1	1	6	5	rs148028531	0.0007∶14795	C = 20/T = 8580	NM_018077.2	c.2273T>C	p.Asp758Gly	40.00%	poss. damaging
chr7∶128037009	*IMPDH1*	1	7	6	5	rs61751223	0.0052∶2280	C = 23/T = 8577	NM_000883.3	c.887T>C	p.His296Arg	40.00%	benign
chr7∶131910932	*PLXNA4*	1	0	1	0	novel	not found	not found	NM_020911.1	c.1970C>T	p.Ser657Asn	40.00%	prob. damaging
chr9∶95155422–95155423	*OGN*	1	0	1	0	novel	not found	not found	NM_014057.3	c.372_373 delAA	p. Leu124fs	50.00%	frameshift
chr20∶34219872	*CPNE1*	1	0	1	1	novel	not found	not found	NM_003915.5	c.547A>T	p.Ser183Thr	66.67%	benign
chr9∶96436037	*PHF2*	1		15	26	rs41276200	0.002∶2389	A = 120/G = 8480	NM_005392.3	c.2519G>A	p.Ser840Asn	N/A	benign
chr3∶38307398	*SLC22A13*	1		20	23	rs72542450	0.01∶1459	A = 84/G = 8516	NM_004256.3	c.47G>A	p.Arg16His	N/A	benign
chrX:140785792	*SPANXE*	1,2		14/7	9/15	novel	not found	not in database	NM_145665.1	c.124G>T	p.Leu42Ile	N/A	not scored

The rare variants common to the two affected individuals were genotyped in 975 cases and 1014 controls. Penetrance with regard to the PD phenotype was assessed in 6 family members belonging to the same generation as the affected individuals. EA = European American.

### Segregation Analysis and Genotyping of Additional *PLXNA4* Variants

The remaining four variants shared by the two affected individuals (Table 1) were pursued further by Sanger-sequencing-based testing for segregation in 6 family members belonging to generation IV. Under the assumption that a given variant would be causal for PD, penetrance ranged between 40.0 and 66.6% in 6 individuals belonging to generation IV. Moreover, on careful scrutiny of the exome data, both index patients were found to harbor one additional, variant of *PLXNA4* (p.Phe40Leu (rs145024048, 111/8489 in NHLBI-ESP exomes) for IV:11 and p.Arg302His (rs143813209, 3/8597 in NHLBI-ESP exomes) for IV:18). These two variants were also genotyped in 15 additional members of the family. *PLXNA4* p.Phe40Leu was found in 5 additional individuals and p.Arg302His was found in 7 additional family members. Importantly and contrary to the exome sequencing data, by Sanger sequencing, IV:11 was also found to harbor the *PLXNA4* p.Arg302His variant. The combination of the *PLXNA4* index variant and p.Phe40Leu was present only in IV:11, while the index variant and *PLXNA4* p.Arg302His were found in a total of 7 individuals belonging to the pedigree. None of the three additional candidate genes harbored additional non-synonymous coding variants in either IV:11 or IV:18.

### Linkage Analysis

In order to further prioritize genes for follow-up, we performed parametric linkage analysis. In doing so, we identified 25 genomic regions with a suggestive linkage signal (LOD≥0.5) (Figure 1B). Only one of these regions, located on chromosome 7 (chr7∶106,254,234 to 134,663,671; maximum two-point LOD score = 0.76), contained one of the four candidate genes identified during exome sequencing, lending further support to the potential causality of variants in *PLXNA4*.

### Mutational Screening of *PLXNA4* in Case/Control Cohort

**Figure 2:**
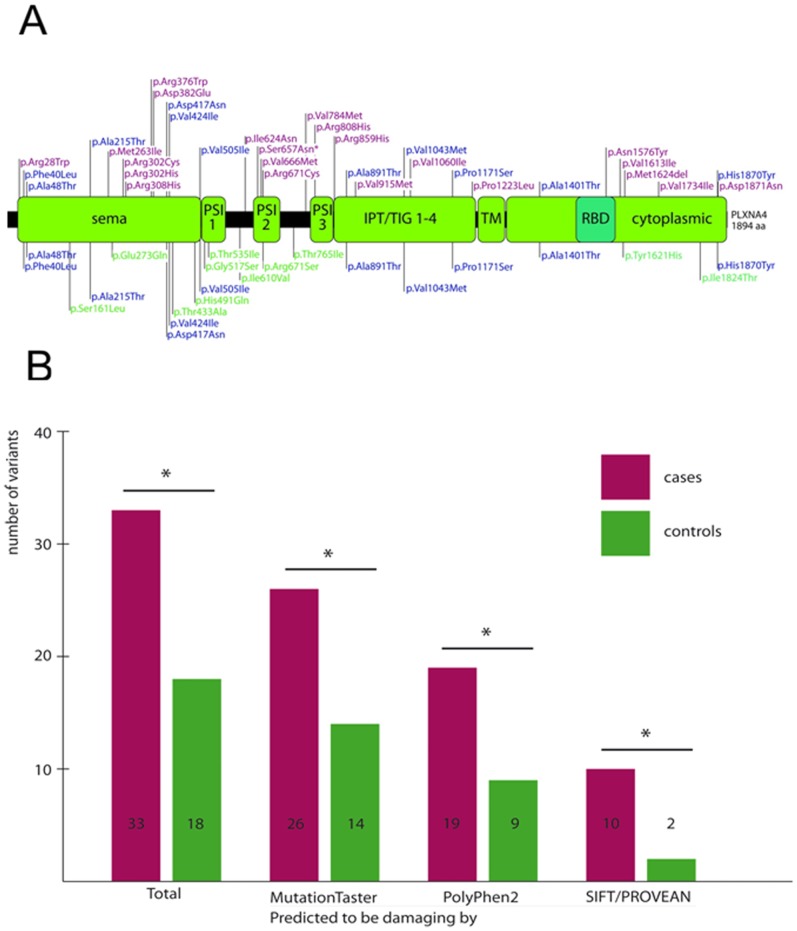
Mutation Screening of *PLXNA4* in PD case/control cohort. (A) Location of *PLXNA4* variants identified in variant screening in relation to known functional domains. An asterisk denotes the variant identified by exome sequencing. blue = variants found in both cases and controls, green = variants found only in cases, purple = variants found only in controls. (B) Analysis of *PLXNA4* variants using SIFT/PROVEAN, PolyPhen2 and MutationTaster reveals an excess of rare non-synonymous variants predicted to be damaging. Insertions and deletions cannot be assessed by PolyPhen2 all and were, therefore, omitted from the analysis using this algorithm.

Linkage analysis highlighted the variant in *PLXNA4* as a potentially causal or modifying variant for the PD phenotype in our family. Also, the affected amino acid in *PLXNA4* is highly conserved in all vertebrates and two of three commonly used prediction algorithms [12]–[14] predicted it to be “damaging”. Accordingly, we screened the 32 coding exons as well as the exon/intron boundaries of *PLXNA4* in 862 Austrian and German cases and 940 controls in order to assess a fuller spectrum of rare genetic variation found. For the most part, this cohort comprised the same individuals used for the above frequency assessment. In *PLXNA4*, a total of 38 novel (37 non-synonymous, 1 deletion) and 6 known variants (rs143813209, rs113830939, rs112682233, rs62622406, rs117458710 and rs73155258, all non-synonymous) resulting in a change in the amino acid sequence were identified (Table S2 in File S1). The large majority (86.21%) of variants were very rare, with MAF≤0.2% in controls. Overall, a similar number of cases (n = 107) and controls (n = 117) harbored at least one variant predicted to result in a changed amino acid sequence (p>0.05, χ^2^ test). The same held true when only variants with MAF≤1.0% (46 cases vs. 52 controls, p>0.05, χ^2^ test) were evaluated. Very rare variants with MAF≤0.2%, however, were more common in cases (n = 33) than controls (n = 18) (p<0.02, χ^2^ test). Three cases but no controls were compound heterozygous for a non-synonymous variant in *PLXNA4*. Variants were located throughout the entire gene (Figure 2A).

Of the individuals harboring a rare non-synonymous variant in *PLXNA4*, information regarding family history was available for 17 individuals: 3 reported a first or second degree relative with PD and a positive history of essential tremor was present in the mother and a maternal uncle in one additional individual. The only brother of the individual harboring the *PLXNA4* p.Arg302Cys amino acid change was also found to have PD and to harbor this variant. However, the family was too small for formal segregation analysis.

When analyzed by means of three commonly used prediction algorithms (PolyPhen2, MutationTaster, SIFT) [12]–[14], the number of non-synonymous single nucleoide variants (SNVs) classified as functionally “damaging” (SNVs classified as “probably damaging” by PolyPhen2, “disease causing” by MutationTaster and “damaging” by SIFT) was greater in cases than in controls. This was especially prominent and statistically significant for PolyPhen2 when only very rare variants with MAF≤0.2% in controls were analyzed (PolyPhen2∶19 variants in cases vs. 9 variants in controls, p = 0.033, χ^2^ test; MutationTaster: 26 in cases vs. 14 in controls, p = 0.028, χ^2^ test; SIFT/PROVEAN: 10 in cases vs. 2 in controls, p = 0.018, Fisher’s Exact test) (Figure 2B). Deletions, which were only found in cases, cannot be assessed by PolyPhen2 and were, therefore, omitted from the analysis using this algorithm.

### Functional Assessment of *PLXNA4* p.Ser657Asn in Fibroblasts

**Figure 3:**
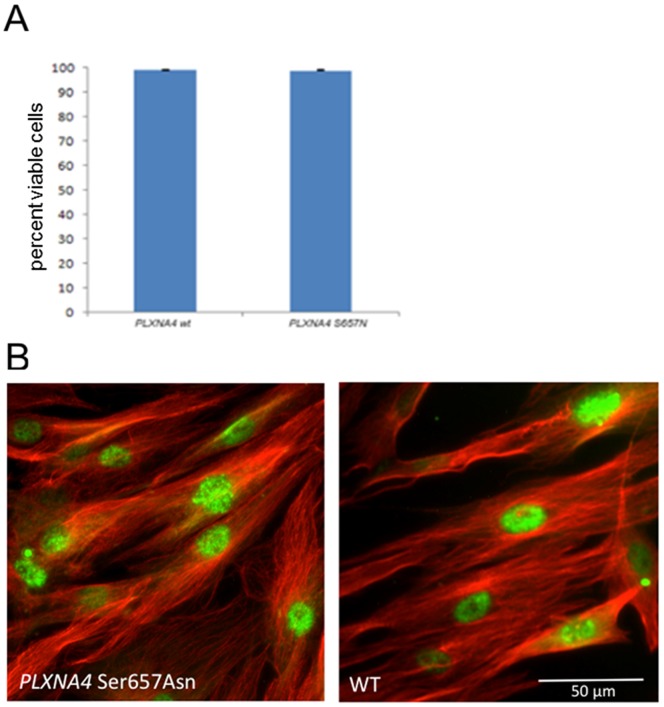
Assessment of cell viability and subcellular protein localization in fibroblasts. (A) The presence of *PLXNA4* p.Ser657Asn do not affect cell viability as assay by live-dead staining and FACS. (B) Immunohistochemistry shows similar subcellular localization of *PLXNA4* (anti-PLXNA4, Sigma, 1∶500) in fibroblasts with and without the p.Ser657Asn amino acid substitution (scale bar = 50 µm).

In fibroblast cell lines generated from both the index patient and an offspring who does not harbor the *PLXNA4* p.Ser657Asn variant (other variants not given to protect privacy) cell viability was similar (Figure 3A). Based on the results from the above mutation screening as well as the fact that *PLXNA4* is known to be expressed in the brain [15] and a role for axonal guidance factors similar to *PLXNA4* already postulated in PD [16], we further analyzed subcellular localization of the protein in the two cell lines but could not detect a difference (Figure 3B).

### Modeling a Potential Role of *PLXNA4* in the PD Network

**Figure 4:**
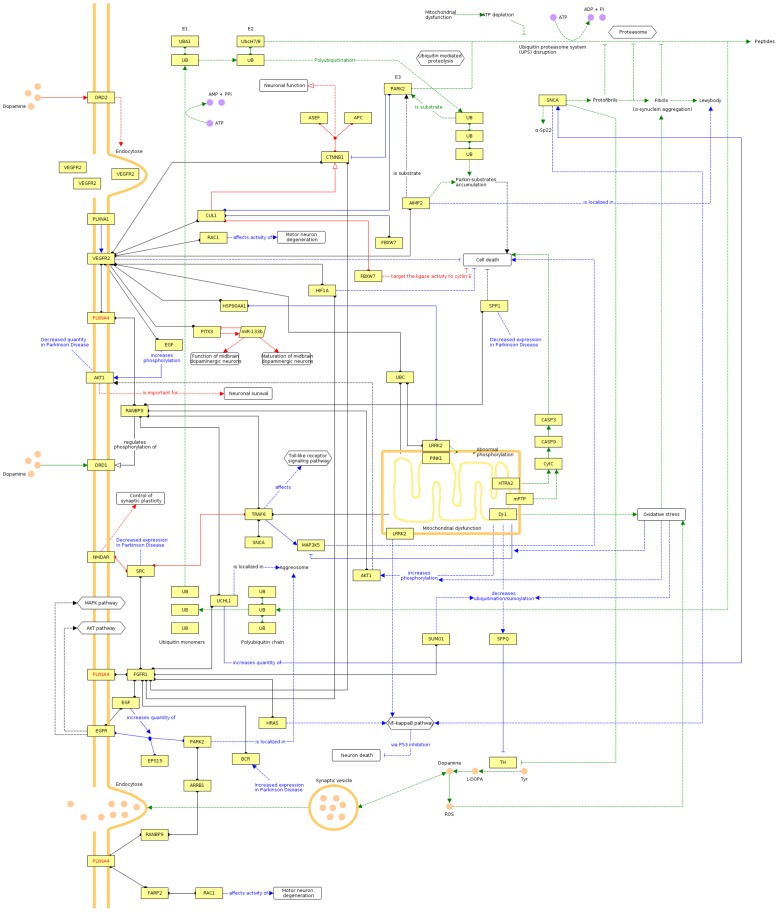
Qualitative multifactorial interaction network of *PLXNA4* and genetic factors with known and hypothetical relevance to PD. Edges obtained from CIDeR are highlighted in blue, PD-specific pathways from KEGG are given in green, red edges denote annotations from OMIM and edges extracted from literature, protein-protein interaction databases or high-confidence predictions are colored black. Undirected protein-protein interactions hold circular ends, directed molecular relations are marked by arcs, whereas general regulations have arrows with no filling, activations have filled arrows and inhibitions have blunted end. Dashed lines indicate indirect effects.

Beyond a proposed general role of axonal guidance pathways in the development of neurodegeneration [16], [17], it is interesting to note that *PLXNA4* can be place into a network containing several firmly established PD genes (*SNCA, PARK2, DJ-1, LRRK2*), although both known and less reliable projected interactions have to be utilized (Figure 4).

## Discussion

In an unbiased, whole-exome approach, we identified a variant in *PLXNA4* (p.Ser657Asn) as a candidate for a potentially causal variant in familial PD. Although this finding is intriguing and functionally plausible, we cannot conclude that this variant in *PLXNA4* is indeed the cause of PD in our family. Also, it is interesting that both affected individuals were found to harbor two or three non-synonymous variants in *PLXNA4*, thus, highlighting the possibility that a “multi-hit” model within the same gene or pathway could play a role with regard to phenotype expressivity.

Three of the final four variants (*PLXNA4* p.Ser657Asn, *OGN* p.L124fs and *CPNE1* p.Ser183Thr) are extremely rare and were only found in other family members but not in approximately 8,978 other individuals of European descent (genotyping sample (n = 1989), in-house exomes (n = 1739), 1000genomes (n = 1000) and NHLBI-ESP exomes (n = 4250)). This is interesting in light of the fact that–with regard to drug target genes–it was recently shown that the rarer a given variant the more likely it is functionally relevant [18]. Yet, on the other hand, this rarity also means that from a genetic standpoint, at the moment, one can neither confirm nor exclude the possibility of a causal or modifying role in the PD phenotype. Further, even taken together additional evidence highlighting *PLXNA4* p.Ser657Asn (suggestive linkage signal, high conservation and predicted pathogenicity, excess of very rare coding variants in cases and functional considerations) can be viewed as suggestive at best and by no means exclude the possibility of other causative or modifying genetic factors that play a role in the PD phenotype in our family.

In general, these findings highlight the fact that in many cases very large populations will be needed to conclusively judge the disease-related nature of a rare variant. Recent studies show that while the power to detect associations for genes harboring rare variants varies widely across genes, only <5% of genes achieved 80% power even assuming high odds ratios (OR) of 5 and when tested in 400 cases and 400 controls. In the same scenario, no gene out of 12,000 genes tested achieved 80% power when assuming an OR of 1.5 [19]. Statistical evaluation is further complicated by the fact that it is not unreasonable to assume that many genes will habor both variants that are protective and predisposing with regard to a given phenotype, as was recently shown for the *APP* locus in Alzheimer’s disease [20], which with the statistical analysis tools available today will always lead to an underestimation of the genetic contribution of rare variants at a given locus to a phenotype’s heritability [21].

Ultimately, it is also possible that the truly causal variant was not picked up in this study because it lies outside the targeted regions of the exome. Here, the use of two enrichment kits of different sizes and different exome target definitions represents a specific weakness of the study. Also, we cannot exclude that IV:18 represents a phenocopy and that the underlying cause of PD in his case is different from that of the other affected individuals in the family. If this were the case, a much larger number of candidate variants than those assessed here could contribute to bringing about the PD phenotype in the examined family.

Moreover, copy number variants, another important player in the full spectrum of genetic variation, could, at the time of study, not yet confidently be assessed in exome sequencing data and were, therefore, not evaluated in our study. Lastly, while suggestive non-significant LOD scores have been used to prioritize variants identified in exome [22] or whole genome [23] sequencing they also harbor the potential for the erroneous exclusion of true positives.

The fact that all four candidate variants were also found in unaffected family members, per se does not contradict potential causality as it is known from other autosomal dominant forms of PD that even among members of a single family, penetrance of known PD mutations can vary widely. Of individuals who harbor the *LRRK2* p.Gly2019Ser mutation, for example, only 28% will develop PD by the age of 59 [24]. Thus, predicted penetrance of the variants identified in our family are in line with what is reported in the literature for other forms of autosomal dominant PD.

Plexin A4, *PLXNA4*, which functions as a receptor for class 3 semaphorins, holds a firmly established role in axon guidance in the development of the central and peripheral nervous systems. For example, PlxnA4 has been shown to restrict inappropriate spreading of mossy fibers within the CA3 region of the murine hippocampus [25], to direct basal dendritic arborization in layer V cortical neurons [26] and sympathetic axons [15], [27] as well as lamination and synapse formation in the outer retina [28] in the mouse.

*PLXNA4* has also been implicated in neurodegenerative conditions. In the discovery stage of a large family-based GWAS assessing low-frequency (MAF≤5%) variants in late-onset Alzheimer’s disease an intronic SNP in *PLXNA4* (rs277484, MAF = 2.0% in 1000genomes) yielded the most significant association signal (p = 9.0×10^−10^). Replication, however, is still ongoing [17]. Similarly, preliminary results have suggested decreased *PLXNA4* expression in the motor cortex of individuals with amyotrophic lateral sclerosis when compared to controls, although the sample size of the study was very limited (n = 5) [29].

*PLXNA4* itself has not previously been implicated in PD. Yet, a number of studies have suggested an involvement of axonal guidance pathways in PD. An early GWAS identified a SNP in semaphorin 5A (*SEMA5A)* as the best association signal [16] and systems biology-based follow-up studies reported an overrepresentation of axonal guidance factors in subthreshold association signals [30] which were shown to predict susceptibility to PD [31]. However, both the association signal and the pathway analysis proved difficult to replicate in other cohorts [32]–[34] which may be due to the fact that as one of the very first GWAS it was not conducted to the current quality standards. Expression studies of different brain regions, on the other hand, have repeatedly found an overrepresentation of differentially expressed axonal guidance pathways in individuals with PD when compared to controls [30], [35]–[37]. Axonal guidance pathways have also been implicated in the proper targeting of dopaminergic neurons from the murine mesencephalon to the ipsilateral striatum [38].

At the moment, both functional and genetic data addressing a role of *PLXNA4* as a PD gene are inconclusive. The identification of additional larger families with PD in which *PLXNA4* p.Ser657Asn or p.Arg302His segregate with the phenotype or the replication of the finding of an excess of very rare variants (MAF≤0.02%) in an independent case/control sample would lend further support to a possible role of modifying or causal variants in *PLXNA4* in PD and to the interesting hypothesis of axonal guidance dysfunction in neurodegenerative conditions.

## Supporting Information

Figure S1 **Filtering scheme for variants identified by exome sequencing in the two affected family members examined.**
(TIF)

File S1 **Supporting Methods and Tables. Table S1 in File S1,** Clinical Phenotype of Affected Individuals in PARK_0005. **Table S2 in File S1,** Non-Synonymous and Indel Variants Identified in Variant Screening of *PLXNA4.*
(DOC)
