# Supplementary figures and images for: Rare Variants in PLXNA4 and Parkinson’s Disease
Source: PLoS One. 2013 Nov 11;8(11):e79145. doi: 10.1371/journal.pone.0079145 (PMC3823607; doi:10.1371/journal.pone.0079145)

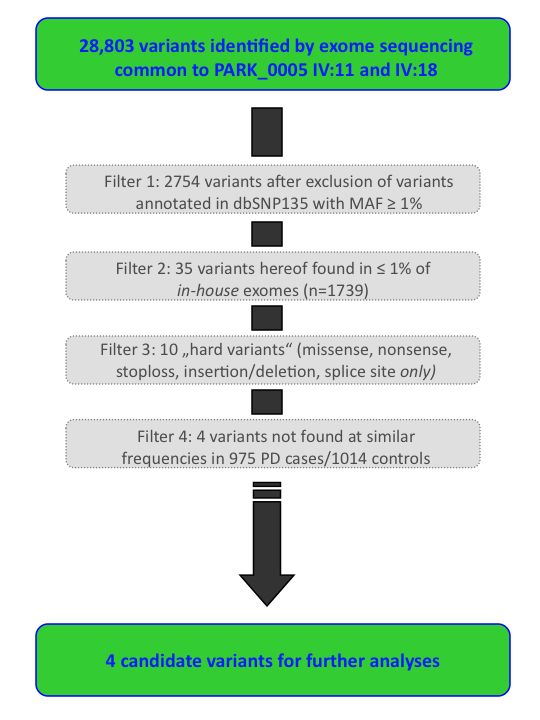

Supplement: Figure S1 — Filtering scheme for variants identified by exome sequencing in the two affected family members examined. (TIF) [file pone.0079145.s001.tif]
