# Supplementary material for: Rare Variants in PLXNA4 and Parkinson’s Disease
Source: PLoS One. 2013 Nov 11;8(11):e79145. doi: 10.1371/journal.pone.0079145 (PMC3823607; doi:10.1371/journal.pone.0079145)
Supplement: File S1 — Supporting Methods and Tables. Table S1 in File S1, Clinical Phenotype of Affected Individuals in PARK_0005. Table S2 in File S1, Non-Synonymous and Indel Variants Identified in Variant Screening of PLXNA4. (DOC) [file pone.0079145.s002.doc]

SUPPORTING INFORMATION

Rare variants in *PLXNA4* and Parkinson’s disease

Eva C. Schulte^a,b^, Immanuel Stahl^a,b^, Darina Czamara^c,d^, Daniel C. Ellwanger^e^, Sebastian Eck^b^, Elisabeth Graf^b^, Brit Mollenhauer^f,g^, Alexander Zimprich^h^, Peter Lichtner^b,i^, Dietrich Haubenberger^h^, Walter Pirker^h^, Thomas Brücke^j^, Benjamin Bereznai^k^, Maria J. Molnar^k^, Annette Peters^l^, Christian Gieger^m^, Bertram Müller-Myhsok^c,d^, Claudia Trenkwalder^f,g^, Juliane Winkelmann^a,b,d,i,n,*^

^a^Neurologische Klinik und Poliklinik, Klinikum rechts der Isar, Technische Universität

München, Munich, Germany

^b^Institut für Humangenetik, Helmholtz Zentrum München, Munich, Germany

^c^Statistical Genetics Unit, Max-Planck Institut für Psychiatrie, Munich, Germany

^d^Munich Cluster for Systems Neurology (SyNergy), Munich, Germany

^e^Chair for Genome-Oriented Bioinformatics, Technische Universität München, Life and Food Science Center Weihenstephan, Freising-Weihenstephan, Germany

^f^Paracelsus Elena Klinik, Kassel, Germany

^g^Neurochirurgische Klinik, Georg August Universität, Göttingen, Germany

^h^Department of Neurology, Medical University of Vienna, Vienna, Austria

^i^Institut für Humangenetik, Technische Universität München, Munich, Germany

^j^Department of Neurology, Wilhelminenspital, Vienna, Austria

^k^Center for Molecular Neurology, Department of Neurology, Semmelweis University,

Budapest, Hungary

^l^Institute for Epidemiology II, Helmholtz Zentrum München, Munich, Germany

^m^Institute for Genetic Epidemiology, Helmholtz Zentrum München, Munich, Germany

^n^Department of Neurology and Neurosciences, Stanford University, Palo Alto, USA

^*^address correspondence to:

Prof. Dr. Juliane Winkelmann, Neurologische Klinik und Poliklinik, Klinikum rechts der Isar, Technische Universität München, Ismaningerstr. 22, 81675 Munich, Germany

Phone: +49 89 4140 4688, Fax: +49 89 4140 4867, Email: winkelmann@lrz.tum.de

**SUPPORTING METHODS**

**Description of case/control sample**

All cases used in variant screening and genotyping were recruited at Paracelsus-Elena Klinik, a hospital specializing in Parkinson’s disease (PD), in Kassel, Germany, as well as at the departments of neurology at Wilhelminenspital and Allgemeines Krankenhaus in Vienna, Austria. The genotyping sample more over included 39 index cases from larger PD families recruited at the Department of Neurology, Semmelweis University, Budapest, Hungary. In all cases, PD diagnosis was made in accordance with the UK Brain Bank Criteria. Controls belong to a large general population cohort (KORA) based in the region around Augsburg in Southern Germany and have been described previously. KORA-AGE represents a subset of the KORA cohort collected in 2009 as a gender- and age-stratified subsample of the KORA S1-S4 studies comprising participants born before 1944 [1]. All individuals taking dopaminergic drugs were excluded from the control sample.

**Demographic data of case/control sample used for frequency assessment**

All ten candidate variants were genotyped in 975 cases (450 of German (age 70.2 ± 9.7yrs, 32.2% female), 486 of Austrian (age 58.7 ±11.3yrs, 35.4% female), and 39 familial cases of Hungarian (age 50.4 ±10.8yrs, 53.8% female) origin) and 1014 population-based controls pertaining to the KORA-AGE cohort (age 76 ± 6.6yrs, 50.1% female).

**Demographic data for case/control sample used for variant screening**

For Idaho®’s LightScanner high-resolution melting curve analysis, 862 cases (376 of German (age 71.1 ± 9.4yrs, 31.6% female), 486 of Austrian (age 58.7 ±11.3yrs, 35.4% female) origin) and 940 population-based controls pertaining to the KORA-AGE cohort (age 76 ± 6.6yrs, 50.4% female) were used.

**Fibroblast cultures**

Skin biopsy samples from the index patient and one of her children were used to generate primary fibroblast cell lines. Cells were cultured in high glucose DMEM with 10% fetal bovine serum (FBS) and 1% penicillin/streptomycin and cultivated at 37 °C in atmospheric oxygen and 5% CO_2_.

**Cell viability**

Cells were staining using a live/dead staining (Invitrogen, aqua) and analyzed by fluorescence activated cell sorting in three independent experiments consisting of one 90% confluent 75cm^2^ flask each for each of the two fibroblast cell lines.

**Immunocytochemistry**

Fibroblasts were plated on coverslips, fixed in 4% paraformaldehyde in growth medium supplemented with 10% FBS, permeabilized with 0.1% TritonX-100 in PBS and stained with rabbit anti-PLXNA4 (1:100, Sigma) in PBS plus 4% FBS. Mouse anti-α-tubulin (1:1000, Sigma) was used to counterstain cells.

**Construction of a qualitative systems biological model**

We collected curated comprehensive protein-protein interactions (PPI) from both high-throughput datasets and individual focused studies from BioGRID (release 3.1.93) [2] and manually annotated protein complexes from CORUM (release February 2012) [3]. Further, the PPI network was extended by known as well as predicted interactions obtained from STRING (version 9.0) [4]. To reduce the noise and thereof to increase the reliability of the model, we required a minimum combined score of 0.4 and additionally a reported high-confidence protein domain interaction in DOMINE (version 2.0) [5]. We integrated the PPI graph over the KEGG (release 64.0) [6] PD pathway (map05012) and the manually curated PD-related interactions from CIDeR (release July 2012) [7]. We extracted all paths of a maximum length of 2 from *PLXNA4* to any KEGG or CiDeR node. The qualitative model was extended by complementary information as annotated in OMIM and found by manual literature search.

**SUPPORTING TABLES**

**Table S1**

Clinical Phenotype of Affected Individuals in PARK_0005.

| individual ID | age at onset | disease duration | IS | B | R | RT | PI | H&Y | L-Dopa/ DA | additional features |
| --- | --- | --- | --- | --- | --- | --- | --- | --- | --- | --- |
| IV:11 | 60 | 15 | B | + | ++ | + | ++ | 3 | ++ | MCI, restless legs syndrome, RBD, hyposmia |
| IV:18 | 67 | 5 | RT | + | + | + | + | 3 | + | MCI, type II diabetes, polyneuropathy, hyposmia |

IS = initial symptom, B = bradykinesia, R = rigor, RT = resting tremor, PI = postural instability, H&Y = Hoehn & Yahr classification, DA = dopamine agonist, RBD = REM-sleep behavior disorder

**Table S2**

Non-Synonymous and Indel Variants Identified in Variant Screening of *PLXNA4.*

| Gene | genomic position | dbSNP132 | variation |  | frequency | | domain |
| --- | --- | --- | --- | --- | --- | --- | --- |
|  | (hg19) |  | nucleotide | amino acid | cases | controls |  |
|  |  |  |  |  | (n=862) | (n=940) |  |
| *PLXNA4* | chr7:132193371 | rs113830939 | c.82 C>T | p.Arg28Trp | 1 |  | sema |
| *PLXNA4* | chr7:132193335 | novel | c.118 T>C | p.Phe40Leu | 35 | 29 | sema |
| *PLXNA4* | chr7:132193311 | novel | c.142 G>A | p.Ala48Thr | 1 | 1 | sema |
| *PLXNA4* | chr7:132192971 | novel | c.482 C>T | p.Ser161Leu |  | 1 | sema |
| *PLXNA4* | chr7:132192810 | novel | c.643 G>A | p.Ala215Thr | 8 | 2 | sema |
| *PLXNA4* | chr7:132192664 | novel | c.789 C>T | p.Met263Ile | 1 |  | sema |
| *PLXNA4* | chr7:132192636 | novel | c.817 G>C | p.Glu273Gln |  | 1 | sema |
| *PLXNA4* | chr7:132192549 | novel | c.904 C>T | pArg302Cys | 1 |  | sema |
| *PLXNA4* | chr7:132192548 | rs143813209 | c.905 G>A | p.Arg302His | 1 |  | sema |
| *PLXNA4* | chr7:132192530 | novel | c.923 G>A | p.Arg309His | 1 |  | sema |
| *PLXNA4* | chr7:132192327 | novel | c.1126 C>T | p.Arg376Trp | 2 |  | sema |
| *PLXNA4* | chr7:132192307 | novel | c.1146 C>A | p.Asp382Glu | 1 |  | sema |
| *PLXNA4* | chr7:132174173 | novel | c.1249 G>A | p.Asp417Asn | 26 | 35 | sema |
| *PLXNA4* | chr7:132174152 | novel | c.1270 G>A | p.Val424Ile | 6 | 4 | sema |
| *PLXNA4* | chr7:132174125 | novel | c.1297 A>G | p.Thr433Ala |  | 1 | sema |
| *PLXNA4* | chr7:131982880 | novel | c.1473 C>A | p.His491Gln |  | 1 | sema |
| *PLXNA4* | chr7:131925916 | rs112682233 | c.1513 G>A | p.Val505Ile | 3 | 5 | sema |
| *PLXNA4* | chr7:131925880 | novel | c.1549 G>A | p.Gly517Ser |  | 2 | PSI1 |
| *PLXNA4* | chr7:131925825 | novel | c.1604 C>T | p.Thr535Ile |  | 1 | PSI1 |
| *PLXNA4* | chr7:131912264 | novel | c.1828 A>G | p.Ile610Val |  | 1 | n/a |
| *PLXNA4* | chr7:131912221 | novel | c.1871 T>A | p.Ile624Asn | 1 |  | n/a |
| *PLXNA4* | chr7:131910932 | novel | c.1970 G>A | p.Ser657Asn* | 1 |  | PSI2 |
| *PLXNA4* | chr7:131908387 | novel | c.1996 G>A | p.Val666Met | 1 |  | PSI2 |
| *PLXNA4* | chr7:131908372 | novel | c.2011 C>T | p.Arg671Cys | 1 |  | PSI2 |
| *PLXNA4* | chr7:131908372 | novel | c.2011 C>A | pArg671Ser |  | 1 | PSI2 |
| *PLXNA4* | chr7:131895706 | novel | c.2294 C>T | p.Thr765Ile |  | 2 | n/a |
| *PLXNA4* | chr7:131888127 | novel | c.2350 G>A | p.Val784Met | 1 |  | n/a |
| *PLXNA4* | chr7:131887568 | novel | c.2423 G>A | p.Arg808His | 1 |  | PSI3 |
| *PLXNA4* | chr7:131887415 | novel | c.2576 G>A | p.Arg859His | 1 |  | IPT/TIG1 |
| *PLXNA4* | chr7:131883311 | rs62622406 | c.2671 G>A | p.Ala891Thr | 26 | 23 | IPT/TIG1 |
| *PLXNA4* | chr7:131878934 | novel | c.2743 G>A | p.Val915Met | 1 |  | IPT/TIG1 |
| *PLXNA4* | chr7:131870089 | rs117458710 | c.3127 G>A | p.Val1043Met | 7 | 14 | IPT/TIG3 |
| *PLXNA4* | chr7:131866930 | novel | c.3178 G>A | p.Val1060Ile | 1 |  | IPT/TIG3 |
| *PLXNA4* | chr7:131865473 | novel | c.3511 C>T | p.Pro1171Ser | 4 | 4 | IPT/TIG4 |
| *PLXNA4* | chr7:131864652 | novel | c.3668 C>T | p.Pro1223Leu | 1 |  | IPT/TIG4 |
| *PLXNA4* | chr7:131853148 | rs73155258 | c.4201 G>A | p.Ala1401Thr | n/a | n/a | cytoplasmic |
| *PLXNA4* | chr7:131833340 | novel | c.4726 A>T | p.Asn1576Tyr | 1 |  | RBD |
| *PLXNA4* | chr7:131832686 | novel | c.4837 G>A | p.Val1613Ile | 1 |  | cytoplasmic |
| *PLXNA4* | chr7:131832662 | novel | c.4861 T>C | p.Tyr1621His |  | 2 | cytoplasmic |
| *PLXNA4* | chr7:131831449-131831451 | novel | c.4872_4874delGAT | p.Met1624_Ile1625delinsIle | 3 |  | cytoplasmic |
| *PLXNA4* | chr7:131829903 | novel | c.5200 G>A | p.Val1734Ile | 1 |  | cytoplasmic |
| *PLXNA4* | chr7:131817926 | novel | c.5471 T>C | p.Ile1824Thr |  | 1 | cytoplasmic |
| *PLXNA4* | chr7:131815315 | novel | c.5608 C>T | p.His1870Tyr | 6 | 15 | cytoplasmic |
| *PLXNA4* | chr7:131815312 | novel | c.5611 G>A | p.Asp1871Asn | | 1 | cytoplasmic |

n/a=not available, * = index variant identified by exome sequencing, PSI=domain found in plexins, semaphorins and integrins, IPT/TIG1= , RBD=RasGAP binding domain

**SUPPORTING REFERENCES**

1. Lacruz ME, Emeny RT, Bickel H, Cramer B, Kurz A, et al. (2010) Mental health in the aged: prevalence, covariates and related neuroendocrine, cardiovascular and inflammatory factors of successful aging. BMC Med Res Methodol 10: 36.
2. Stark C, Breitkreutz BJ, Chatr-Aryamontri A, Boucher L, Oughtred R, et al. (2011) The BioGRID Interaction Database: 2011 update. Nucleic Acids Res 39: D698-704.
3. Ruepp A, Waegele B, Lechner M, Brauner B, Dugner-Kaltenbach I, et al. (2010) CORUM: the comprehensive resource of mammalian protein complexes—2009. Nucleic Acids Res 38: D497-501.
4. Szklarczyk D, Franceschini A, Kuhn M, Simonovic M, Roth A, et al. (2011) The STRING database in 2011: functional interaction networks of proteins, globally integrated and scored. Nucleic Acids Res 39: D561-568.
5. Yellaboina S, Tasneem A, Zaykin DV, Raghavachari B, Jothi R, et al. (2011) DOMINE: a comprehensive collection of known and predicted domain-domain interactions. Nucleic Acids Res 39: D730-735.
6. Kanehisa M, Goto S, Sato Y, Furumichi M, Tanabe M. (2012) KEGG for integration and interpretation of large-scale molecular data sets. Nucleic Acids Res 40, D109-114.
7. Lechner M, Höhn V, Brauner B, Dugner I, Fobo G, et al. (2012) CIDeR: multifactorial interaction networks in human diseases. Genome Biol 13: R62.
